# 

**Published:** 1986-10

**Authors:** 


### Contents

**101 Appreciating Hypertension, Quicksands and Firm Ground**
D. W. Barritt

**105 Assessment of patient's opinions on personalised advice sheet given before they leave the ward after a myocardial infarction**
C. Burns-Cox, P. Davies and S. Teague

**106 Do we tell our coronary patients enough?**
C. Trelawney-Ross and S. C. Jordan

**109 Percutaneous Coronary Angioplasty, the Bristol experience**
S. Aldridge, M. Papouchado, P. Walker, J. Vann-Jones, P. Wilde

**114 From our Foreign Correspondent**

**Health Care in China today**

A. Williams

**116 Book Reviews**
**The Citadel of the Senses,** by McDonald Critchley
**The Urinary Tract and the Catheter,** by Norman Slade and William Gillespie
**Pathology in Surgical Practice,** by G. J. Hadfield, M. Hobsley and B. C. Morson
**Sea Marks,** by John Naish

